# Supplementary material for: Does Chinese-style margin trading promote the high-quality development of listed companies?
Source: PLoS One. 2023 Apr 27;18(4):e0284947. doi: 10.1371/journal.pone.0284947 (PMC10138208; doi:10.1371/journal.pone.0284947)
Supplement: Attachment — Submitted filename: Response to Reviewers.docx [file pone.0284947.s002.docx]

**Response to Reviewers**

Dear Academic Editor and Reviewers:

Thank you for your letter and the reviewers’ comments on our manuscript entitled "Does Chinese-style Margin Trading Promote the High-quality Development of Listed Companies？" (ID: PONE-D-23-03601). Those comments are very helpful for revising and improving our paper, as well as the important guiding significance to other research. We have studied the comments carefully and made corrections which we hope meet with approval. The main corrections are in the manuscript and the responds to the journal requirements and the reviewers’ comments are as follows (the replies are highlighted in blue).

Response to Journal Requirements

Q1: Please ensure that your manuscript meets PLOS ONE's style requirements, including those for file naming.

Response: We modify our manuscript according to PLOS ONE's style guidelines and articles already published by PLOS ONE.

Response to Reviews

Reviewer #1: The Absract meets all requirements. Introduction has been crafted good. Literature review integrates theory. Anaysis is detailed. Discussion is fine. I highly appreciate your effort. All I can recommend is proof reading and addition some latest references. Good luck.

Response: Thanks for your helpful recommendations and your high compliment. We are trying our best to make it better. We add some new literatures into paper, as follows:

1. Bennett B, Stulz R, Wang R. Does the stock market make firms more productive?. Journal of Financial Economics. 2020; 136(2): 281-306. doi: 10.2139/ssrn.3080957
2. Chen S, Lee D. Small and vulnerable: SME productivity in the great productivity slowdown. Journal of Financial Economics. 2023; 147: 49-74. doi: 10.1016/j.jfineco.2022.09.007
3. Dai PY, Yang SG, Yuan L. How does stock liquidity affect total factor productivity? On the resource allocation function of the capital market and its development of marketization and legalization. Statistical Research (Chinese). 2022; 39(9): 62-73. <https://kns.cnki.net/kcms/detail/detail.aspx?dbname=CJFD2022&filename=TJYJ202209004&dbcode=CJFD>
4. Hall BH. The financing of research and development. Oxford Review of Economic Policy. 2002; 18(1): 35-51. doi: 10.1093/oxrep/18.1.35

Reviewer #2: This paper explores whether margin trading promotes high quality growth of listed companies, using a quasi-natural experiment of margin trading reform in the Chinese stock market, and provides some insights into the optimization of the institutional environment of the capital market. The statistical analysis is appropriate, and the empirical results support the hypothesis, but the background and the significance of the research question has not been explained clearly. The mechanism should be studied to reveal the effect further.

Response: Thank you for your wise counsel. Your suggestions have greatly influenced our research and writing.

We made numerous changes to the introduction in order to demonstrate the research background and significance of this paper as clearly as possible (see the first and second paragraphs of the introduction). The support of capital markets is inextricably linked to China's economy's high-quality development. And China's capital market is at a critical stage of reform. Margin trading has been a contentious reform measure in China's stock market in recent years. As a result, the impact of margin trading reform on listed companies' TFP and its mechanism must be clarified. This is not only an intuitive evaluation of the performance of existing reform measures, but also an important reference for future reform measure optimization and improvement. This paper, in particular, closely links the margin trading reform with the TFP of enterprises in theory, based on the stock market information environment and the financing behavior of listed companies. And provides adequate empirical evidence.

In addition, we reorganized the mechanism and presented it succinctly in the introduction. Because of the flaws in reform measures and the unique investment environment of China's stock market, Chinese-style margin trading encourages speculation while discouraging arbitrage. This makes the stock market information environment deteriorate and asymmetric information increase. According to asset pricing theory, the company's market value is reduced, the expected rate of return and the cost of equity capital are increased, and listed companies' external equity financing is hampered. At the same time, according to the principal-agent theory and the dividend signal theory, shareholders have a sufficient incentive to increase cash dividends and reduce retained earnings and internal financing. The concurrent deterioration of internal and external financing will have a negative impact on the TFP of publicly traded companies.

In addition, we have modified and optimized the text expression in other parts of this article based on your suggestions.

We sincerely hope that you will accept our explanation.
